# Influence of Physicochemical Properties of Budesonide Micro-Suspensions on Their Expected Lung Delivery Using a Vibrating Mesh Nebulizer

**DOI:** 10.3390/pharmaceutics15030752

**Published:** 2023-02-23

**Authors:** Katarzyna Dobrowolska, Andrzej Emeryk, Kamil Janeczek, Radosław Krzosa, Michał Pirożyński, Tomasz R. Sosnowski

**Affiliations:** 1 Faculty of Chemical and Process Engineering, Warsaw University of Technology, Waryńskiego 1, 00-645 Warsaw, Poland; 2 Department of Pulmonary Diseases and Children Rheumatology, Medical University of Lublin, A. Gębali 6, 20-093 Lublin, Poland; 3 Allergy and Pulmonology Department, Postgraduate Center for Medical Education, Marymoncka 99/103, 01-813 Warsaw, Poland

**Keywords:** nebulization, aerosol, budesonide, vibrating mesh nebulizer, physiochemical characteristics

## Abstract

The efficiency of lung drug delivery of nebulized drugs is governed by aerosol quality, which depends both on the aerosolization process itself but also on the properties of aerosol precursors. This paper determines physicochemical properties of four analogous micro-suspensions of a micronized steroid (budesonide, BUD) and seeks relationships between these properties and the quality of the aerosol emitted from a vibrating mesh nebulizer (VMN). Despite the same BUD content in all tested pharmaceutical products, their physicochemical characteristics (liquid surface tension, viscosity, electric conductivity, BUD crystal size, suspension stability, etc.) are not identical. The differences have a weak influence on droplet size distribution in the mists emitted from the VMN and on theoretical (calculated) regional aerosol deposition in the respiratory system but, simultaneously, there is an influence on the amount of BUD converted by the nebulizer to aerosol available for inhalation. It is demonstrated that the maximum inhaled BUD dose is below 80–90% of the label dose, depending on the nebulized formulation. It shows that nebulization of BUD suspensions in VMN is sensitive to minor dissimilarities among analogous (generic) pharmaceutics. The potential clinical relevance of these findings is discussed.

## 1. Introduction

Budesonide (BUD) is one of the most used inhaled corticosteroids. It has been available since the 1990s in several inhaled pharmaceutical forms, including aqueous micro-suspension for nebulization [1,2,3,4,5]. Nebulization is a preferred method of BUD delivery, especially in children and neonates. BUD suspensions were initially tested and approved by the FDA for nebulization in the constant-output pneumatic (jet) nebulizers, such as the Pari LC-Jet Plus with the Pari Master compressor [6]. Nowadays, such formulations are often atomized in various pneumatic nebulizers, however, the efficiency and safety of drugs delivered from different nebulizers has been insufficiently studied [7]. During the last 20 years, many new nebulization devices and systems have emerged, including vibrating mesh nebulizers (VMNs) [8,9,10]. VMNs have become very popular, and most of the new drugs are being tested with this type of nebulizer [10]. The advantages of VMNs are: (i) low drug losses, (ii) narrow droplet size distribution in the aerosol cloud, and (iii) high predicted lung deposition [10]. However, there are still not too many pre-clinical and clinical studies of BUD micro-suspensions delivered from VMNs indexed in the PubMed database [11,12,13,14,15,16], and many of such results are available only as abstracts and short communications [17,18,19,20]. The important difference in nebulizing BUD is that physicochemical properties of steroid suspensions are different from typical drugs available as solutions [18], and a thorough recognition and understanding of this process are needed to optimize the pharmacological recommendations, especially when using VMNs.

Therefore, this study is focused on the quantitative assessment of the physicochemical properties of selected BUD suspensions available on the market (including generics), followed by testing efficiency of their nebulization in a selected VMN and numerical evaluation of the regional deposition of generated aerosols in the respiratory system. It should be stressed that the aim of the study is not to compare these pharmaceutical products, but rather to see to what extent their physicochemical and aerosol properties are similar and how this can influence the expected delivered dose.

## 2. Materials and Methods

The BUD products used in the study were coded as A, B, C, and D. Their compositions are listed in Table 1, and additional data can be found in the Supplementary Material. All formulations contain the same declared mass of micronized BUD crystals insoluble in water and are supplemented by adjuvants needed to adjust pH, tonicity, and to extend the product shelf-life. During experiments, the suspensions were evacuated from the original packaging by squeezing the plastic ampoules after gentle manual agitation according to the information provided in the leaflet of the drug packages. Physicochemical properties of the suspensions and their atomization in the nebulizer were characterized with a variety of methods, which are summarized in Table 2 and described in detail in the following sections.

### 2.1. Micro-Suspension Stability and Electrokinetic Data

**Figure 1:**
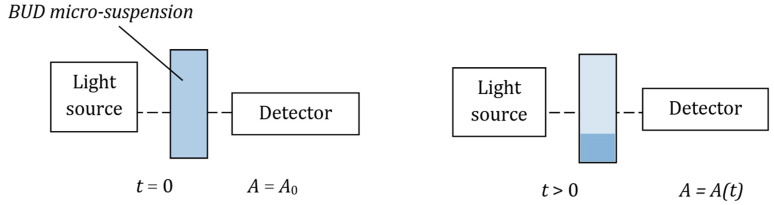
The principle of the spectrophotometric measurement of micro-suspension stability. *A* denotes the light absorbance: *A(t)* < *A*_0_.

The stability of BUD suspensions was studied by measuring time changes in the optical absorbance of light, *A* (λ = 540 nm), using Genesys 10S UV-VIS spectrometer (Thermo Scientific, Waltham, MA, USA). The measurements were performed in 1 mL disposable polyacrylic cells. The overview of the experiments is shown in Figure 1. According to the proposed technique, the absorbance measured at 5 mm above the cell bottom is expected to decrease faster in suspensions with lower stability (i.e., when BUD particles sediment quicker). Each BUD suspension was analyzed in triplicate and the results were averaged.

Electrokinetic properties of drug suspensions (the ζ-potentials) were measured using the Zetasizer Nano ZS (Malvern, Worcestershire, UK). Due to the high electrical conductivity of the original drug products caused by the high concentration of electrolytes (mainly NaCl), the measurements had to be performed after drug dilution with MilliQ water (Merck Millipore, Burlington, MA, USA). In addition to ζ-potential, the conductivity and pH of the samples were also determined. The experiments were performed in triplicate and the results were averaged.

### 2.2. Particle Size and Morphology

The BUD particle size (volume-based diameter, *dv*) was determined for 10 mL samples of each drug using the laser diffraction particle analyzer LS 13 320 XR (Beckman Coulter Inc., Brea, CA, USA; range: 0.01–3500 μm) equipped with the Universal Liquid Module. The measurements were performed in two variants: in the raw samples and in the samples after sonication by sonotrode of the analyzer. Each measurement was performed in triplicate and the results were averaged.

Crystal size and morphology were also observed at 10,000× magnification with the scanning electron microscope (SEM-Hitachi TM1000, Tokyo, Japan). A washing procedure was required before the observations to remove all soluble additives which otherwise would crystallize on the particles after water evaporation. Pure BUD crystals were obtained by repeated centrifugation (3 min at 10,000 rpm) and washing with MilliQ water, and finally followed by drying in filtered air under atmospheric pressure. Before the observations, dried crystals were coated with a 25 nm layer of gold with the K-550X sputter coater (Quorum Technologies, Lewes, UK) for improving SEM pictures’ quality.

### 2.3. Surface Tension and Rheological Properties of the Drugs

Surface tension of drug samples was determined with the pendant drop technique using the PAT-1M tensiometer (Sinterface, Berlin, Germany), based on the changes in the shape of 15 μL droplet formed at the tip of a steel capillary. The measurements were performed at 25 ± 0.5 °C during the first 600 s after droplet formation, and this allowed us to determine both the dynamic (*σ*_d_) and the static (quasi-equilibrium, *σ*) surface tension. The data for each drug were measured in triplicate and averaged. Rheological properties of all BUD suspensions were measured by plate–plate rheometry using MCR102 (Anton Paar, Graz, Austria) at 25 ± 0.5 °C, applying the shear rate, $\dot{\gamma }$, of 10–100 s^−^^1^. Each sample was measured in triplicate and the data were averaged. Since pharmaceutical suspensions may be, in general, non-Newtonian [21], the apparent viscosity, $\mu_{app}$, was determined as:

(1) $$\mu_{app}=f\left(\dot{\gamma }\right)=\frac{\tau }{\dot{\gamma }}$$

where τ denotes the shear stress (Pa). For Newtonian liquids, $\mu_{app}$ is equivalent to the dynamic viscosity.

### 2.4. Nebulization Rate and Droplet Size Distribution

**Figure 2:**
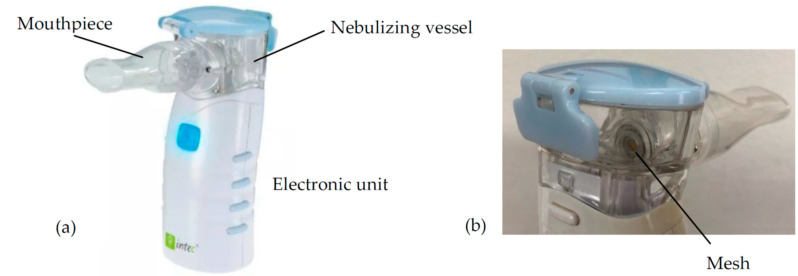
Intec Twister Mesh NE-105 nebulizer (VMN): (**a**) general view and (**b**) close-up of the nebulizing vessel (rear view).

BUD suspensions were nebulized in the battery-operated VMN Intec Twister Mesh NE-105 (Intec Medical, Cracow, Poland) shown in Figure 2. Micrometer-size droplets are formed in this device by forcing the liquid out the reservoir vessel through micrometer-sized orifices in the polymeric membrane, which vibrates with ultrasonic frequency (100 kHz) [22,23]. The details of the VMN, including the structure of the mesh, are provided in the Supplementary Material.

Liquid medications are aerosolized in this device independently of the patient breath. Nebulized liquid is not recycled (i.e., it is atomized only once when it passes the mesh), unlike in jet nebulizers where the drug is recirculating due to liquid drainage after separation of large droplets impacting on baffles [24].

The nebulization rate was measured gravimetrically, by weighing the nebulizing vessel at 1 min intervals during 8 min of the nebulization process, with aerosol emitted to the waste. Although this is not the standard (compendial) method of nebulizer testing, it is well-suited for the purpose of this study, allowing to determine the maximum aerosol mass output of the VMN with various formulations. Each BUD suspension was studied in three independent experiments and the data were averaged.

Droplet size distribution in the aerosol emitted from the VMN was analyzed using the Spraytec^®^ diffraction spectrometer (Malvern, Worcestershire, UK) operated in the open-bench configuration. This technique has been proven to be alternative to the impactor-based methods of nebulizer testing [25,26] and it has been successfully used for nebulizer evaluation [27,28]. Spraytec^®^ allows determining the volume-based droplet size distribution (*Dv* in the range of 0.1–900 μm) directly at the aerosol source, i.e., just after droplets leave the nebulizer. This eliminates the problem of droplet evaporation during the measurements using impactors [29]. The aerosol was sampled at a 1 Hz frequency for 10 s, and then the data were averaged over time. Based on the complete droplet size distribution, the basic numerical parameters were evaluated: the median diameter (*Dv50*), the tenth centile (*Dv10*), and the Span, showing the width of the distribution:

*Span = (Dv90 − Dv10)/Dv50* (2)

Each BUD formulation was studied in triplicate and the results were averaged.

### 2.5. Estimation of Regional Droplet Deposition in the Respiratory Tract

The deposition of aerosol droplets in different regions of the respiratory tract was calculated based on the measured droplet size distribution using the MPPD (multi-path particle deposition) model [30]. The calculations were performed for a symmetrical lung with the Yeh-Schum geometrical parameters [31], assuming oral tidal breathing (12 breaths per minute, breath volume 500 mL), with the standard values of the functional residual capacity (FRC = 3300 mL) and the volume of upper airways (50 mL).

### 2.6. The Emitted BUD Dose during Nebulization

**Figure 3:**
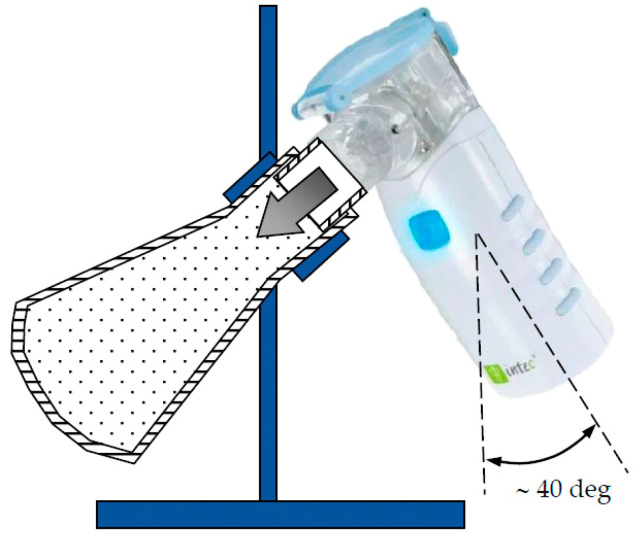
Scheme of the aerosol collection system.

The emitted dose of BUD was measured by collecting the total amount of aerosol released from the nebulizer. BUD suspension was transferred from an ampoule to the nebulizer and 1 mL of demineralized water was added to the ampoule to recover BUD that might remain in the ampoule after squeezing. Then, the nebulizer outflow tube was fitted to the neck of a 250 mL Erlenmeyer flask using parafilm. The flask was positioned at approximately 40–45 degrees to avoid droplet retention on the nebulizer outflow tube (Figure 3). It has been verified that this tilt of the nebulizer did not affect aerosol generation. It has also been confirmed that because the VMN does not cause airflow (like jet nebulizers), such collection is efficient and free of aerosol loss. After nebulization had stopped, 1 mL of demineralized water was added to the nebulizer and then the nebulization was continued to aerosolize any BUD that might remain on the walls of the nebulizing vessel. This aerosol was also collected in the Erlenmeyer flask. After complete condensation of the aerosol, the inner walls of the flask were washed with 2 mL of methanol, and the full content of the vessel was transferred to a 10 mL volumetric flask. Then, the Erlenmeyer flask was washed twice with 1 mL methanol aliquots, and the liquid was transferred to the volumetric flask. Finally, the volumetric flask was filled up to 10 mL with methanol. Each formulation (A, B, C, and D) was tested in 9 independent trials. To clean the VMN after each nebulization experiment, the nebulizing vessel was washed with 2 mL of demineralized water, and then an additional 2 mL of water was used to clean the mesh by pumping water in the opposite direction (i.e., from the outlet tube into the nebulizing chamber), using so-called reverse cleaning [22,23]. After washing, the nebulizer chamber was allowed to dry before the subsequent measurement.

The amount of BUD in each collected sample was determined by ultra-high-performance liquid chromatography (UPLC) using the UPLC Waters Acquity system with the Acquity PDA eλ liquid chromatography/mass spectrometer (LC/MS) detector (Waters Corporation, Milford, CT, USA) [32]. The system was equipped with an Acquity UPLC BEH C18 column (100 mm × 2.1 mm, 1.7 μm) with the pre-column Acquity UPLC BEH C18 VanGuard (5 mm × 2.1 mm, 1.7 μm). The complete description of the analytical procedure is provided in the Supplementary Material. Statistical tests were performed with Statistica software (v. 13, Dell, distributed by Statsoft Polska, Cracow, Poland). The regression curve determined from the responses for standard solutions was used to determine the BUD concentration in the tested samples. The goodness of fit was verified with the Mandel test, and the distribution of the residuals was analyzed with the Shapiro–Wilk test. Due to the large number of data obtained for the nebulized samples, the uniformity of the results was evaluated with one-way and two-way ANOVA tests. The differences were analyzed with post-hoc Tukey analysis. The final results were compared using Kruskal–Wallis analysis. A *p*-value of less than 0.05 was considered statistically significant.

## 3. Results

### 3.1. Microsuspension Stability and Electrokinetic Data

**Figure 4:**
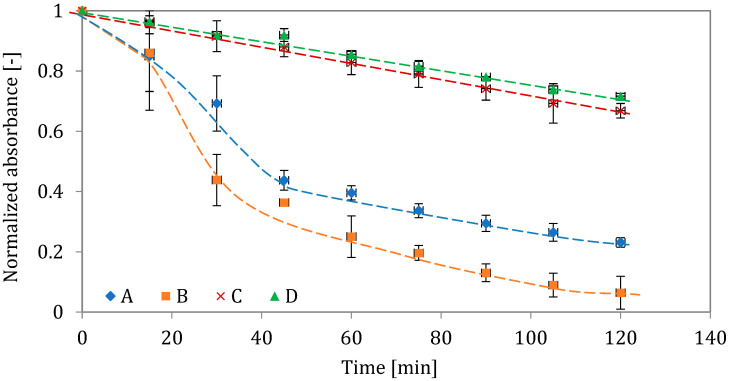
Stability of BUD suspensions A, B, C, and D, as illustrated by the relative change in the light absorbance. Error bars show the standard deviation (*n* = 3). Lines are drawn to guide the eye.

Figure 4 shows the change in the normalized light absorbance (*A*/*A*_0_) measured in all tested BUD suspensions. According to these data, suspensions C and D are more stable than A or B. The relatively fast reduction in the absorbance in suspension A and even faster in suspension B suggested the sedimentation of BUD microcrystals, i.e., weaker stability, which may result from the larger size of BUD particles, also including the formation of agglomerates. Zeta-potential (ζ) was in the range between −6.5 and −3 (Table 3), indicating no notable differences in the adhesive interactions between BUD particles regardless of the sample. On the other hand, the ionic content (expressed by conductivity) of the studied products was slightly different, which can result in variable electrostatic interactions in BUD suspensions. The differences in conductivity were statistically significant (<0.05) for the samples but not for products A and B (*p* = 0.47).

### 3.2. BUD Particle Size and Morphology

**Figure 5:**
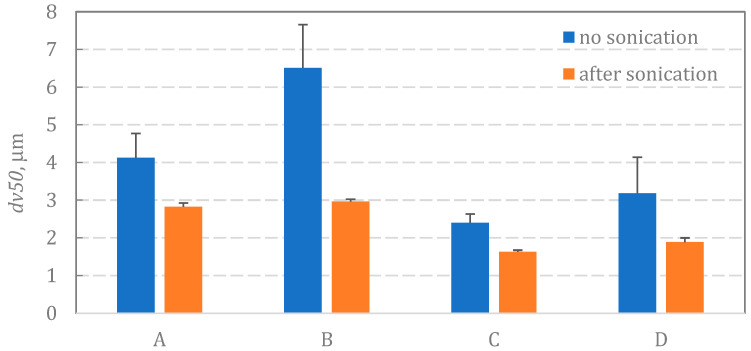
Median diameter of crystal size before and after sonication.

The data of BUD crystal size (volume-based diameter: *dv*) obtained from the laser diffraction measurement are presented in Figure 5, indicating the variation in the crystal size among the studied suspensions. These data show the influence of the hydrodynamic stresses on the break-up of particle clusters. Particles formed the largest agglomerates in suspension B (median diameter, *dv50* = 6.5 μm) and A (*dv50* = 4.1 μm), whereas the clusters were smaller in suspensions C and D (2.4–3.2 μm). Differences in crystal size for unsonicated suspensions were statistically significant (*p* < 0.05), except for the differences between suspensions A and B (*p* = 0.06), A and D (*p* = 0.31), and C and D (*p* = 0.33). These results correspond to the results of suspension stability (Figure 4), where BUD particles in samples A and B sedimented faster than in C or D. After particle de-agglomeration caused by ultrasounds applied to samples in the particle size analyzer (which can be compared to the situation during ultrasonic vibrations in VMNs), BUD particles in C and D became smaller (*dv50* = 1.6–1.9 μm) than in A (*dv50* = 2.8 μm) and B (*dv50* = 3.0 μm). All differences in the size of crystals after sonication were statistically significant (*p* < 0.05), except for the difference between suspensions A and B (*p* = 0.17). This difference in crystal size may have an influence on the amount of drug passing through the micro-apertures of the vibrating mesh of the nebulizer.

**Figure 6:**
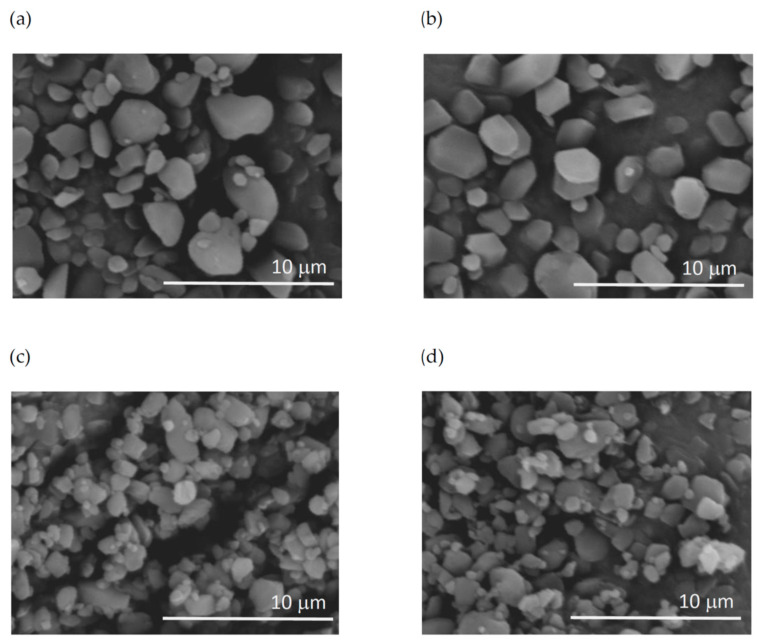
SEM pictures of BUD particles in the suspensions (**a**–**d**) (magnification ×10,000).

SEM pictures of BUD microparticles (Figure 6) confirmed that particles present in suspensions A and B were larger than those in suspensions C and D. Particles found in A and B were more regular in shape and many of them had the size of ~3 μm, whereas no particles larger than 2 μm were visible in samples C and D. Suspensions C and D also contained many sub-micrometer-sized crystals, which were absent in suspensions A and B.

### 3.3. Surface Tension and Rheological Properties of the Drug

Table 4 shows the comparison of the quasi-equilibrium (static) surface tension of the tested BUD suspensions (σ), measured by the pendant drop method 600 s after the formation of a new air/liquid interface. Since BUD is not soluble in water and not surface-active [33], the observed reduction in the surface tension of 0.9% saline suggests that probably, the adjuvants used in BUD nebulization products (see Table 1) were responsible for lowering the surface tension. As seen in Table 4, σ was slightly lower in samples C and D (~40 mN/m) than in A (~42 mN/m) or B (~42.5 mN/m). The differences in the surface tension were statistically significant (*p* < 0.05), but not for product pairs A and B (*p* = 0.48) and C and D (*p* = 0.68). The variability in σ may be considered insignificant, as the surface tension of all BUD products was almost 30 mN/m lower than that of water. However, these differences may be considered not essential, noting that the surface tension of each BUD product was almost 30 mN/m lower than that of water.

**Figure 7:**
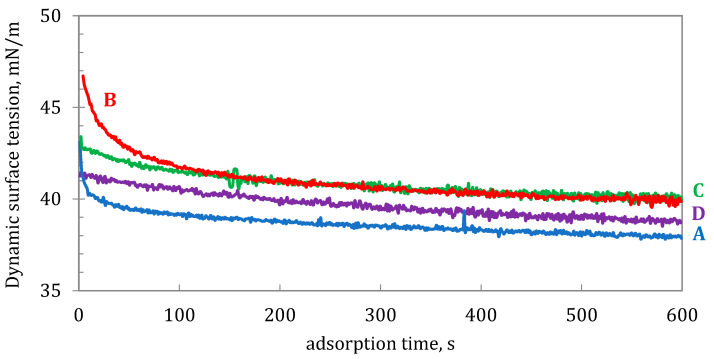
Dynamic surface tension, ***σ****_d_*, of BUD suspensions A, B, C, and D (25 ± 0.5 **°**C).

Theoretically, low surface tension should result in smaller droplets emitted from VMNs, although this dependency can be more complicated due to some individual features of each device [34,35,36]. On the other hand, it is probable that the dynamic (σ_d_) rather than the equilibrium (σ) surface tension is a more important parameter in the nebulization since the new interfacial area of generated droplets is formed very quickly. Under such conditions, there is not enough time for effective adsorption of surface-active molecules at the newly formed air/liquid interface, so the actual surface tension in the system is higher than σ [37]. The relationships of σ_d_ vs. time for the studied BUD suspensions are plotted in Figure 7, showing that BUD suspensions differed not only in quasi-equilibrium surface tension (listed in Table 4) but also in σ_d_, measured in single seconds. The graph also shows different rates of surface tension reduction in the samples. The role of this phenomenon for the droplet formation process in VMNs probably needs further clarification.

**Figure 8:**
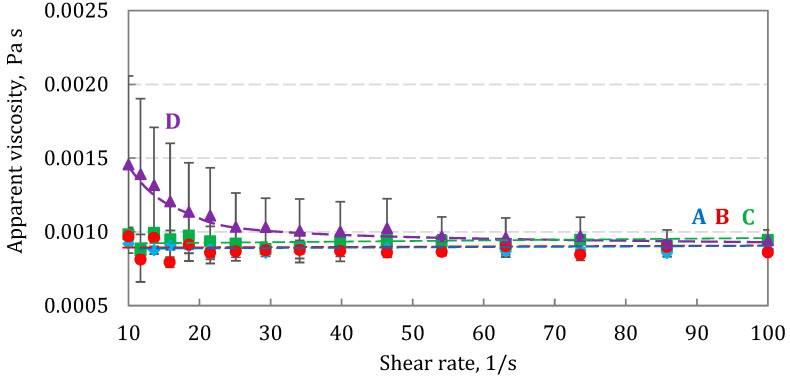
Apparent dynamic viscosity as a function of shear rate for BUD suspensions A, B, C, and D (25 ± 0.5 °C). Error bars show the standard deviation (*n* = 3). Lines are drawn to guide the eye.

Figure 8 compares the apparent viscosity as a function of shear rate, $\dot{\gamma }$ (see Equations (1) and (2)), for all BUD formulations. Three suspensions (A, B, C) were Newtonian in the studied range of $\dot{\gamma }$, with *μ*_app_ close to 1 mPa s. In contrast, the apparent viscosity of suspension D was higher at low shear rates (i.e., for $\dot{\gamma }$ < 30 s^−1^) and then was reduced to approximately 1 mPa s. This can be explained by the presence of particle clusters, which de-agglomerate after applying a shear rate. However, the relatively large scatter of these data also suggests that this clustering may be rather incidental, and the differences were not statistically significant. Considering that nebulization is a process accompanied by high shear rates, it can be expected that the viscosity of the suspension should have no effect on aerosol generation.

### 3.4. Atomization Rate and Droplet Size Distribution and Estimation of Regional Deposition of Inhaled Aerosol Droplets in the Respiratory Tract

**Figure 9:**
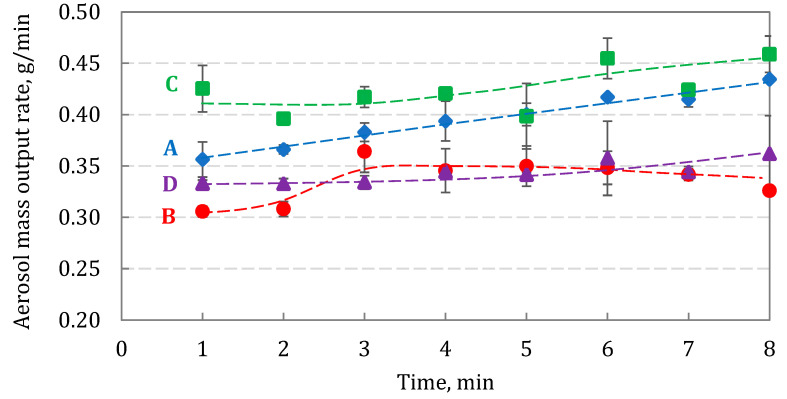
Aerosol mass output rate of BUD suspensions A, B, C, and D. Error bars show the standard deviation (*n* = 3). Lines are drawn to guide the eye.

The total aerosol mass output rate vs. the nebulization time is shown in Figure 9, while time-averaged values are summarized in Table 5.

**Figure 10:**
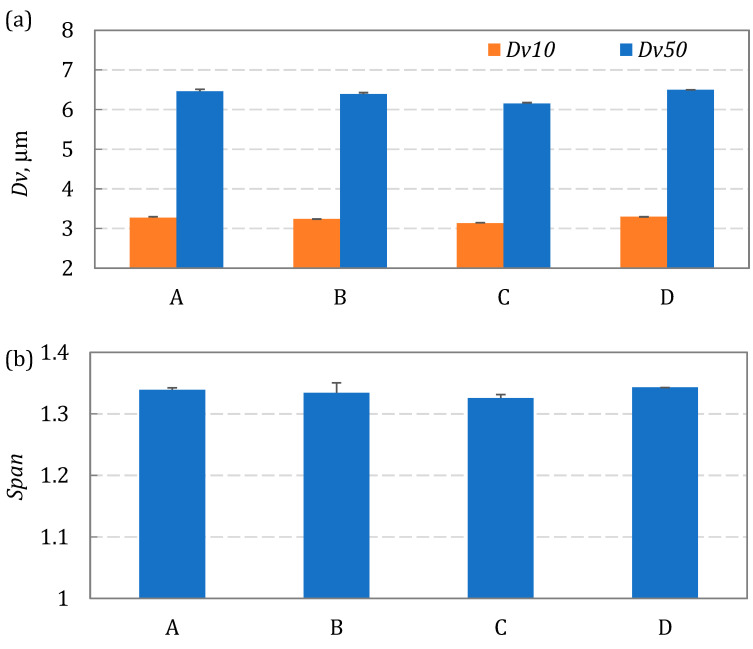
(**a**) Volumetric median diameter, *Dv50,* and volumetric diameter *Dv10* of emitted droplets. (**b**) *Span* of the distribution. Error bars show the standard deviation (*n* = 3).

The average output rate was slightly different, with the highest value for suspensions C (0.424 ± 0.023 g/min) and B (0.336 ± 0.021 g/min). For all drugs, the output slightly increased with the nebulization time. It can be explained by the increase in the liquid temperature during atomization with ultrasound vibrations, which influenced the suspension properties. Although the increase in temperature in VMNs was much smaller than in the standard ultrasonic nebulizers driven by higher ultrasound frequencies (1–2 MHz) [34], it still lowered both the viscosity and the surface tension of BUD suspensions. This should result in more efficient pumping of liquid through the mesh micro-apertures and thus easier droplet formation. Figure 10a,b compare the values of the median volumetric diameter, *Dv50*, *Dv10*, and *Span* of aerosols emitted from the nebulizer for all BUD suspensions. *Dv10* is the droplet diameter corresponding to 10% of the cumulative volume (that is, also the mass) of all droplets, so it indirectly informs about the size of the smallest droplets in the emitted cloud, which allows to assess whether they can carry BUD crystals (see the Section 4). *Dv10* and *Dv50* data are highly reproducible for each suspension (SD in the range of 0.01–0.05 μm), as is *Span* (SD < 0.03).

As seen in Figure 10, 10% of the mass of the liquid forming the aerosol was contained in droplets smaller than 3.1–3.3 μm, which excludes the possibility of emitting some BUD crystals inside them. There was also virtually no difference in droplet size distribution among BUD suspensions, so their similar penetration and deposition in specific parts of the respiratory system can be expected. The regional deposition of inhaled aerosol in the respiratory system calculated using the MPPD model is shown in Table 6. The variability of deposition for each BUD suspension was always below 0.3% due to the high reproducibility of *Dv50* for each suspension.

For each formulation, 45–46% of the aerosol can be delivered to the lower respiratory tract. It may be noted, though, that these predictions neglect the fact that the smallest inhaled droplets may not contain BUD, so the real amount of delivered drug can be lower than that predicted by the computations. Inertial deposition of large droplets in the oropharynx is in the range of ~29.5–30% for formulations A, B, and D, and slightly lower (27%) for formulation C. Low deposition in the mouth and throat is beneficial for low local side-effects of inhaled corticosteroids [38].

### 3.5. BUD Doses

Data in Table 7 show the comparison of the average BUD doses present in the ampoules (with the nominal dose of 1000 μg), denoted as the actual dose, *AD_BUD_*, and the mass present in the emitted aerosol, denoted as the nebulized dose, *ND_BUD_*.

It is interesting to note that *AD_BUD_* recovered from the ampoules was larger than the nominal dose (101.8–102.6% on average), which may be due to the manufacturers’ tolerance of volumetric filling of the ampoules with a liquid drug. The average nebulized doses, *ND_BUD_*, were more product-dependent, and they were in the range of 77.7% of the nominal dose (suspension B) to 89.1% (suspension D). The percentage of residual BUD dose remaining in the nebulizing vessel after complete nebulization (*RF_BUD_*) was calculated as:

*RF_BUD_ = (AD_BUD_ − ND_BUD_)/AD_BUD_ × 100%* (3)

which is in the range of 13.2–24.0%, indicating incomplete emission of BUD from the nebulizer for each suspension. These *RF_BUD_* values were not related to the residual volume of this VMN (which was less than 0.2 mL, i.e., 10% of the initial drug volume in the studies), since BUD that might remain in the nebulizer was nebulized again after adding additional water aliquots (see Section 2).

Kruskal–Wallis analysis confirmed statistically significant differences in BUD content in the collected aerosol (γ^2^(4) = 63.89; *p* < 0.001). Post-hoc Tukey analysis (Table 8) showed that the average BUD dose in the nebulized aerosol of formulation C was statistically significantly higher than in the case of formulation B (*p* < 0.001) and comparable to formulations A and D (*p* = 1.00 and *p* = 0.59, respectively). The average BUD dose nebulized from formulation D was statistically significantly higher than from formulation B (*p* < 0.001) and comparable to formulation A (*p* = 0.91). Finally, the average nebulized BUD dose collected in formulation B was statistically significantly lower than in formulation A (*p* < 0.001).

## 4. Discussion

The study investigated the effect of BUD suspension properties on the characteristics of aerosol generated in the VMN and predicted lung deposition. The four commercial BUD inhalation micro-suspensions tested with identical nominal BUD concentrations showed some differences regarding physicochemical properties. They were related to:

1. Differences in the surface tension (static and dynamic), ionic strength, and particle sedimentation rate, which can be attributed to the unequal concentrations of the adjuvants used to tune the suspension pH, isotonicity, and stability, and to extend the product shelf-life (see Table 1).
2. Different size distribution of BUD crystals in the suspensions.

Both factors may seem to be unimportant assuming that the quality of generic drugs is fully assured only by the mass of the pharmaceutical active ingredient (here, BUD) forming the dose. However, drugs delivered by inhalation from nebulizers must be effectively converted to inhalable aerosol, and differences in the physicochemical properties of the pharmaceutical product can influence the aerosol quality and hence the drug delivery [34,35,36,37,39,40,41]. It is known that inhaled aerosol particles or droplets must be smaller than 5 µm to reach the small bronchi, bronchioles, and the pulmonary region, whereas the size of 5–10 µm helps to target the oropharynx [42,43]. VMNs (but also other types of medical nebulizers) are designed to generate a large fraction of droplets smaller than 5 µm, which is typically verified by nebulization of NaCl or NaF solutions [44,45].

**Figure 11:**
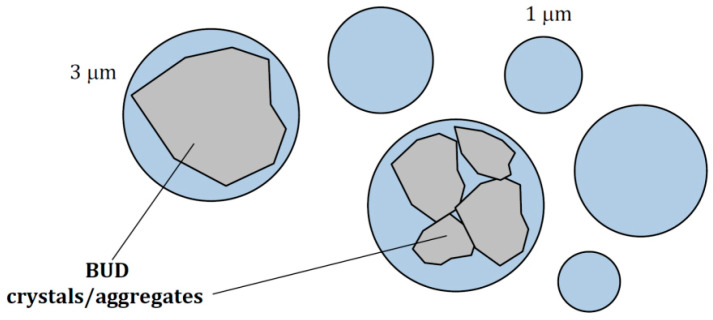
Possible geometric limitations for BUD particles/aggregates contained inside aerosol droplets (droplet diameter 1–3 µm). The smallest droplets may contain no BUD particles.

When the liquid is a suspension, insoluble drug microparticles must be carried inside the droplets emitted from the nebulizer. This means that the size of crystals but also their tendency to form stable agglomerates are essential for the quality of suspensions for inhalation (Figure 11).

Differences in particle size and agglomeration may be due to different processing of BUD particles (e.g., during powder micronization) before they are used to produce individual BUD nebulization suspensions [46]. Our results showed that two of the suspensions (C and D) contained smaller BUD particles than the other two (A and B), and this has consequences for suspension stability. This is illustrated in Figure 4, Figure 5 and Figure 6 by the results of particle sedimentation rate, measured crystal size distribution, and SEM observations of BUD particles. Even with ultrasounds, such as those generating hydrodynamical stresses inside VMNs, differences in particle size are still maintained. The use of VMNs for nebulization of suspensions is even more problematic as the liquid must pass the micro-apertures in the mesh during aerosol formation. The polymeric mesh of the tested Twister Mesh NE-105 VMN contains conical apertures with the narrowest diameter around 3 µm (see Supplementary Material), which limits the emission of larger BUD crystals and their aggregates. As a result, these largest BUD particles will remain in the nebulizer [47], whereas some of the droplets emitted by the VMN will contain only the solvent (i.e., saline with soluble adjuvants). This scenario was indirectly confirmed by our results, showing that the amount of BUD emitted from the VMN was lower by 13–24% than the amount used for nebulization (Table 7). These results also showed statistically significant differences between the tested formulations. If some crystals do not exit the nebulizer, they also cause a gradual increase in the BUD concentration in the suspension, favoring crystal aggregation and increasing the viscosity of the suspension [48]. These phenomena are expected to reduce the rate of nebulization [49,50,51], although our results did not confirm this (Figure 9). This can be explained by the simultaneous increase in the temperature of the liquid in the nebulizing vessel (because of ultrasonic vibrations), which counteracts the above-mentioned changes in suspension properties caused by higher BUD concentrations. An important problem related to the larger size of BUD crystals is the possibility of clogging the mesh of the VMN [52,53]. This effect is reduced in devices that are equipped with the ‘reverse cleaning system’ (as in the VMN used in this work), which protects the orifices in the mesh from permanent contamination [22,23].

Another issue, although not discussed here, is the velocity of the aerosol emitted from the nebulizer, which also influences the amount of drug delivered to the lungs. The results obtained with the laser Doppler analysis (LDA) [54] allow to evaluate the role of droplet impaction on the elements attached to the nebulizer, such as the mouthpiece, mask, or the tubing of the nebulizer–ventilator circuit. Such losses of the drug have a fundamental role, e.g., in the effective treatment of patients under ventilatory support [55,56].

There is only scarce literature on the nebulization of BUD formulations that can be directly related to our results. Some innovative liquid BUD nanosuspensions were studied and compared to the commercial products [45,57,58,59], indicating the potential role of the formulation for BUD delivery efficiency and efficacy. It must be noted, though, that proposed changes in the formulation and physicochemical characteristics of BUD inhalation products should be safe regarding interactions of inhaled and deposited aerosols in the pulmonary region [60]. Differences in physiochemical properties of drug formulations were also reported for other generic drugs delivered as aerosols. It was shown that several analogous liquid nasal formulations of the glucocorticosteroid (mometasone furoate 50 μg/mL), delivered as a spray by nasal pumps, had different surface tension and rheological characteristics. These properties influenced the spraying characteristics and regional deposition in the model nasal cavity [21], which confirms that the issues pointed out in this paper also exist in other liquid formulations used as precursors of aerosol drugs.

It is arguable whether the 10–15% difference in the BUD total dose delivery shown in the current study for similar nebulization products has clinical implications [59]. It is known that for inhalation drugs, the target (delivered) dose depends on physicochemical and patient-related factors. Large inter-subject variability of aerosol delivery is well-documented and is caused by variations in the inhalation technique and airway morphology, both being dependent on the patient’s age and health status. Therefore, it is not possible to precisely predict the actual delivered dose of the nebulized drug to specific regions of the respiratory system of a given patient, particularly when potential drug losses during aerosol uptake are ignored [56]. According to EMA regulations, if the emitted drug aerosol is kept within a given range compared to the reference drug (the originator), it is assumed to be therapeutically equivalent [61,62]. These regulations do not require to demonstrate the similarity in other parameters of the formulations nor do they require testing the product with specific nebulizers. As shown here, preserving only an equal amount of API in nebulization drugs, especially for suspensions, may not guarantee full reproduction of the aerosol dose delivered from any nebulizer. Our results at this stage of research (i.e., without in vivo data) can only be taken as an indication of some of the overlooked issues regarding suspensions aerosolized in nebulizers, which should be taken into account in the development of new or generic drugs.

## 5. Conclusions

Budesonide micro-suspensions are formulations widely used in the treatment of inflammatory diseases of the lower respiratory tract using nebulization. The presented study demonstrated that some differences in the physicochemical properties (viscosity, static and dynamic surface tension, crystal size and morphology, etc.) exist among the equivalent BUD products for nebulization with the same nominal content of the corticosteroid. In two of the four products, the primary BUD particles were larger (d*v50* = 2.5–3 μm) than in the other two (*dv50* = 1.6–1.9 μm), and there was a difference regarding the size of BUD aggregates (*dv50* = 4.1–6.5 μm vs. 2.4–3.2 μm) and the stability of the suspension, as defined by the sedimentation rate of the particles. Despite some variations in surface tension and other physicochemical properties, the nebulizer mass output rate and aerosol droplet size distributions for all BUD formulations were nearly the same. Therefore, the calculated deposition efficiency of inhaled aerosol in the lower airways was similar (45–46%) and only minor differences were predicted in aerosol deposition in the oropharynx (27–30%). However, an important result of this study is that approximately 10% by weight of BUD suspensions nebulized in this VMN was contained in droplets smaller than ~3 μm. This size is comparable with the median size of BUD crystals in two suspensions, so it is probable that up to 10% of aerosol droplets emitted from these products may contain no BUD. This was partly confirmed by the observed differences in the mass of emitted BUD aerosol, which was in the range of 77.7–87.8% of the nominal dose (1 mg of BUD) for two suspensions containing larger BUD particles and in the range of 87.6–89.6% of the nominal dose for two suspensions containing smaller particles. It may be concluded that some BUD particles were retained in the nebulizer because they were not able to pass the micro-apertures in the mesh.

The study clearly showed that additional factors must be considered in the design and evaluation of the nebulization process using drug suspensions as compared to solutions of soluble drugs. Nebulizing devices, in particular VMNs, should be carefully matched with the formulation properties, considering the proper crystal size, crystal stability against aggregation, and other physicochemical parameters to be sure that these factors do not change the expected outcome of inhalation therapy.

## Figures and Tables

**Table 1:** Composition of BUD suspensions (data according to the product leaflets).

BUD Suspension	A	B	C	D
Active ingredient	budesonide (0.5 mg/mL)	budesonide (0.5 mg/mL)	budesonide (0.5 mg/mL)	budesonide (0.5 mg/mL)
Additives ^1^:				
EDTA Na_2_	+	dihydrate	+	+
NaCl	+	+	+	+
polysorbate 80	+	+	+	+
citric acid	+	monohydrate	+	+
solvent	purified water	water for injection	water for injection	water for injection

^1^ Concentrations not specified. EDTA Na_2_—Edetate disodium salt.

**Table 2:** Types of experiments for sample characterization.

Type of Experiment	Method/Equipment
Suspension stability	UV spectrometry: GENESYS 10S UV-Vis (Thermo Scientific, Waltham, MA, USA)
Electrokinetic properties (ζ-potential, conductivity, pH)	Zetasizer Nano ZS (Malvern, Worcestershire, UK), conductivity and pH-meter (Elmetron, Zabrze, Poland)
Dynamic and static surface tension	Pendant drop tensiometry: PAT-1M (Sinterface, Berlin, Germany)
Rheological characteristics	Plate–plate rheometry: MCR 102 (Anton Paar, Graz, Austria)
Nebulizer mass output	Gravimetry: analytical balance (Radwag, Radom, Poland)
BUD particle morphology and size	Scanning electron microscopy: TM-1000 (Hitachi, Tokyo, Japan)
BUD particle size distribution	Laser diffraction in liquid: LS 13 320 XR (Beckman Coulter, Brea, CA, USA)
BUD mass-delivered from the nebulizer	Ultra-high-performance liquid chromatography (UPLC) (Waters, Milford, CT, USA)
Aerosol droplet size distribution	Laser diffraction in air: Spraytec (Malvern, Worcestershire, UK)

**Table 3:** Electrokinetic characteristics of the BUD formulations (SD = standard deviation, *n* = 3). Original samples were diluted due to a too-high electrolyte concentration.

BUD Suspension	BUD Concentration in the Sample (mg/mL)	pH	Conductivity (mS/cm)		Zeta Potential (mV)	
			κ	SD	ζ	SD
A	0.0313 *	~7	3.85	0.15	−4.32	0.43
B	0.0625	~7	3.98	0.17	−3.14	0.14
C	0.0625	~7	2.66	0.06	−6.26	0.31
D	0.0625	~7	4.84	0.29	−4.70	0.77

* More diluted because of too-high electrolyte concentration for the reliable *ζ* measurement.

**Table 4:** Quasi-equilibrium (10 min) surface tension of tested BUD suspensions at 25 ± 0.5 °C.

BUD Suspension	Quasi-Equilibrium Surface Tension *σ*, mN/m	SD mN/m
A	41.9	0.6
B	42.6	1.1
C	40.2	0.1
D	40.3	0.3

**Table 5:** The average mass output (aerosol emission) rate of BUD suspensions in the VMN. SD denotes the standard deviation (*n* = 3).

BUD Suspension	Output Rate, g/min	SD, g/min
A	0.396	0.027
B	0.336	0.021
C	0.424	0.023
D	0.344	0.011

**Table 6:** Calculated deposition of inhaled aerosol droplets in different regions of the respiratory system (MPPD model, standard inhalation conditions via mouth).

BUD Suspension	Deposition Efficiency			
	Oropharynx (Mouth and Throat)	Trachea + Bronchi	Pulmonary Region	Total in the Lower Airways
A	29.4%	25.0%	20.0%	45.0%
B	29.9%	25.1%	19.8%	44.9%
C	27.0%	24.9%	21.2%	46.1%
D	29.9%	25.1%	19.8%	44.9%

**Table 7:** Amounts of BUD in the inhalation products (before and after nebulization) and calculated mean residual amounts. The nominal (label) BUD dose in all formulations was 1000.0 μg.

BUD Suspension	Actual BUD Dose, *AD_BUD,_* μg Average ± SD	Nebulized BUD Dose, *ND_BUD,_* μg Average ± SD	Average Residual BUD Mass in Relation to Actual Dose, *RF_BUD_,* %
A	1018.2 ± 20.5	875.0 ± 60.6	14.1
B	1023.5 ± 14.2	777.4 ± 48.6	24.0
C	1024.2 ± 27.0	875.7 ± 38.5	14.5
D	1025.6 ± 20.2	890.5 ± 19.9	13.2

**Table 8:** Statistical significance of the results (*p*-value, post-hoc Tukey analysis).

	A	B	C
B	<0.001	-	<0.001
C	1.00	<0.001	-
D	0.91	<0.001	0.59

## Data Availability

Additional data related to this study are available on request from the corresponding author.

## Supplementary Materials

The following are available online at https://www.mdpi.com/article/10.3390/pharmaceutics15030752/s1, Table S1: Characteristics of Intec Twister Mesh NE-105 nebulizer, Table S2: Results of regression analysis, Figure S1: SEM pictures of both sizes of the mesh. References [22,23,32,43,63,64,65] are cited in the supplementary materials.
