# Supplementary material for: Influence of Physicochemical Properties of Budesonide Micro-Suspensions on Their Expected Lung Delivery Using a Vibrating Mesh Nebulizer
Source: Pharmaceutics. 2023 Feb 23;15(3):752. doi: 10.3390/pharmaceutics15030752 (PMC10059591; doi:10.3390/pharmaceutics15030752)
Supplement: Supplementary file 1 [file pharmaceutics-15-00752-s001.zip › pharmaceutics-2141646-supplementary.pdf]

pharmaceutics

MDPI

Supplementary Material

S1. Nebulizer characteristics

The essential data of Intec Twister Mesh NE-105 nebulizer are listed in Table S1. SEM pictures of both sizes of the mesh are shown in Figure S1.

Table S1. Characteristics of Intec Twister Mesh NE-105 nebulizer [S1, S2].

Feature	Details
Mesh vibration frequency	100 kHz
Mesh material	polymeric (not specified)
Mesh diameter and shape	2 mm, dome-like
Number of apertures	175
Aperture diameters (conical shape)	3 µm (min) – 30 µm (max) - Figure S1
Distance between apertures	70 µm - Figure S1
Declared volumetric liquid output (for saline)	0.25 mL/min
Residual volume	below 0.2 ml
Manufacturer	Foshan Ganuying Electronics Co., Ltd., Guandong, China
Power supply	2AA batteries or 3V DC
CE Mark	YES

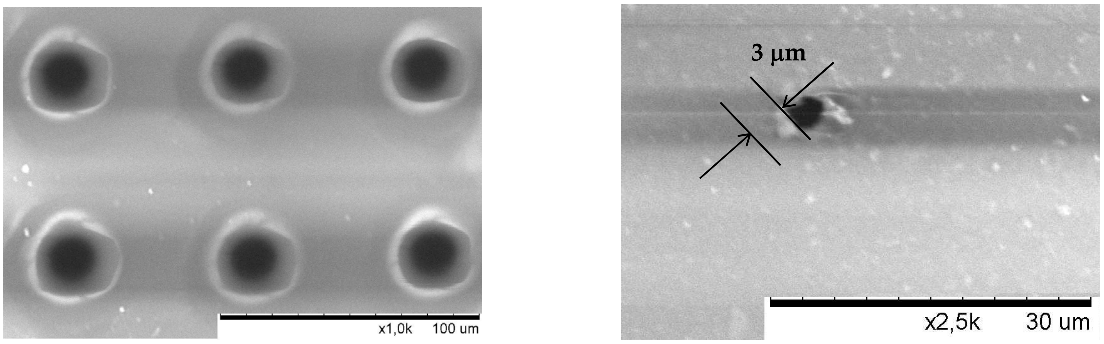

Figure S1. SEM pictures of both sizes of the mesh (TM-1000 SEM, Hitachi, Japan). The minimum aperture size is approximately 3 µm (right panel). The diameter of the conical aperture on the other side of the mesh is approximately 30 µm (left panel).

S2. Budesonide inhalation products.

This scientific research was not aimed at determining advantages of any pharmaceutical product. Four BUD micro-suspensions available in Poland were used in the studies for checking the similarity of their physicochemical and aerosol properties as the quality and therapeutic equivalence of each pharmaceutical product had been assessed by the regulatory agency EMA according to the known requirements [S3, S4]. The following products were used in the study (listed in alphabetical order): Benodil® 1 mg/2 mL (Polpharma S.A., Starogard Gdański, Poland), Budixon Neb® 1 mg/2 mL (Adamed Pharma S.A., Pieńków, Poland), Nebbud® 1 mg/2 mL (Teva Pharmaceuticals Polska, Warsaw, Poland), Pulmicort® 1 mg/2 mL (AstraZeneca AB, Sodertalje, Sweden).

S3. Analytical procedures for BUD determination with UPLC and data analysis

Analytical determinations were done at the Department of Medicinal Chemistry, Jagiellonian University Medical College, Kraków, Poland. Stock solution of BUD was prepared by dissolving 1 mg standard of BUD in methanol to the final concentration of 1 mg/mL. The solution was stored at -20 °C until preparation of standard solutions. Stock solution of internal standard (chloramphenicol - CHL) was prepared from 10 mg of chloramphenicol dissolved in methanol to the final concentration of 1 mg/mL. The solution was stored at -20 °C until the preparation of standard solutions. Standard solutions of BUD were prepared using 400 μL of stock solution of BUD (1 mg/mL) mixed with water-methanol mixture (1:1, v/v) up to 1 mL total volume. Next, 500 μL of this solution was diluted accordingly three times. Each solution was mixed with 100 μL of stock solution of chloramphenicol and filled up with methanol to 1 mL. This allowed to obtain the final BUD solutions with the concentrations 25 μg/mL, 50 μg/mL, 100 μg/mL and 200 μg/mL, and 100 μg/mL of chloramphenicol. These solutions were prepared on the same day when the sample analysis was performed. Stock solutions of commercial BUD products was prepared by transferring the full content of an ampoule (after a gentle manual agitation) to a volumetric flask (10 mL). Next the ampoule was washed twice with 1 mL of water. Methanol was added to the collected mixture in the flask to obtain the final volume of 10 mL.

Quantitative analysis of BUD was done in the ultra-high-performance liquid chromatography (UPLC) using UPLC Waters Acquity system with ACQUITY PDA eλ liquid chromatography/mass spectrometer (LC/MS) detector (Waters Corporation, Milford, MA, USA) [S5]. The system was equipped with Acquity UPLC BEH C18 column (100 mm x 2.1 mm, 1.7 μm) with a pre-column Acquity UPLC BEH C18 VanGuard (5 mm x 2.1 mm, 1.7 μm). Analysis was done using a linear gradient of eluent A (100% to 0% in 2 minutes), then pure eluent B for 1 min, and next a linear gradient of eluent A (0 to 100% in 1 min at 0.3 ml/min flow). Eluent A was water (0.1% HCOOH v/v), eluent B was acetonitrile (0.1% HCOOH, v/v). Absorbance was measured by PDA (photodiode array) detector for 200-700 nm wavelength scanned with 1.2 nm resolution with a rate of 10 points/sec. Standard solution samples were analyzed using six 2 μL injections. Samples from nebulization were determined using three 2 μL injections. High quality materials were used: methanol and acetonitrile for LC/MS (Sigma-Aldrich, Poznan, Poland), budesonide (purity ≥99%, Sigma-Aldrich, Poznan, Poland), chloramphenicol (purity ≥98%, Sigma-Aldrich, Poznan, Poland). Filtered (0.2 μm) demineralized water (conductance < 0,2 μS/cm) was obtained from the demineralizer (model HLP5 - Hydrolab, Straszyn, Poland). The concentration was calculated from PDA chromatograms that show the sums of absorbance in the wavelength range. The area under the peaks of chloramphenicol (residence time, RT = 2.03 min) and of both BUD stereoisomers (RT = 2.47 min) were determined. The raw data were elaborated with MassLynx V4.1 software (Waters Corporation, Milford, MA, USA). After that, the response was calculated as:

$$\text{Response} = AUC_{BUD} \times C_{CHL} \times AUC_{CHL} \qquad (S1)$$

where AUC_{BUD} – area under the peak of BUD, C_{CHL} – chloramphenicol concentration (100 μg/mL), AUC_{CHL} – area under the peak of chloramphenicol.

The UPLC/MS analysis confirmed that the relationship between the AUC for both BUD diastereoisomers and the concentration could be described by the parabolic function and the correlation of these parameters was statistically significant ($p<0.0001$; $r^2>0.99$). The Mandel test proved that parabolic model was better than linear one ($p<10^{-6}$). The residuals of linear model had the normal distribution ($p>0.65$). The parameters of calibration curve are listed in Table S2.

Table S2. Results of regression analysis.

Parameter	Value
a2	-0.00079 ± 0.00001 ($p<10^{-6}$)
a1	0.84161 ± 0.02330 ($p<10^{-6}$)
a0	-2.97844 ± 0.98190 ($p<0.007$)
r	0.9995
r^2	0.9989
Test of normality of Shapiro-Wilk residues	$p>0.65$
Mandel fit test	$p<10^{-6}$
% of recovery	101.51 ± 0.67

Neither the limit of detection (LOD) or the lower limit of quantification (LLOQ) were not determined for the method as BUD concentrations analyzed in these studies were high (in the order of 0.1 mg/mL). According to literature data, the LOD and LLOQ for budesonide in UPLC/MS analysis is 1 ng/mL [S6], and in modern tandem systems it cen be even below 5 pg/mL [S7].

References

S1. Twister Mesh NE-105 – operation manual (in Polish). Available online: https://www.intecmedical.pl /inhalator-membranowy- siateczkowy-intec-twister-mesh.html (accessed on 6 December 2022).
S2. Aura Portable Nebulizer – operation manual. Available online: https://www.aura-medical.com/wp-content/uploads/2019/05/ AuraMedical_A6_32_portaneb_Notforprinting.pdf (accessed on 6 December 2022).
S3. Pirożynski, M.; Sosnowski, T.R. Inhalation devices: from basic science to practical use, innovative vs generic products. *Expert Opin Drug Del* **2016**, 13, 1559-1571.
S4. CHMP. Guideline on the pharmaceutical quality of inhalation and nasal products. European Medicine Agency, London, 2006.
S5. ACQUITY UPLC Photodiode Array and e*λ* Photodiode Array Detector, Operator's Overview and Maintenance Guide. Available online: https://www.waters.com/webassets/cms/support/docs/715002209ra.pdf (accessed 6 December 2022).
S6. Szeitz, A., Manji, J., Riggs, K.W., Thamboo, K., Javer, A.R. Validated assay for the simultaneous determination of cortisol and budesonide in human plasma using ultra high performance liquid chromatography–tandem mass spectrometry, *J. Pharm. Biomed. Anal.* **2014**, 90, 198-206.
S7. Rao, B.T., Mantha, S., Vaidyanathan, G. Quantification of budesonide using UPLC and Xevo TQ-S. Application Note, Waters Corp. Available online: https://www.waters.com/content/dam/waters/en/app-notes/2012/720004198/720004198-es.pdf (accessed 15 February 2023)
